# Fire-induced structural changes and long-term stability of burned historical rag papers

**DOI:** 10.1038/s41598-018-30424-7

**Published:** 2018-08-13

**Authors:** Kyujin Ahn, Andreas Schedl, Thomas Zweckmair, Thomas Rosenau, Antje Potthast

**Affiliations:** 1 University of Natural Resources and Life Sciences, Vienna, Department of Chemistry, Division of Chemistry of Renewable Resources, Muthgasse 18, 1190 Vienna, and Konrad Lorenz Straße 24, A-3430 Tulln, Austria; 2 Archival Preservation and Restoration Center, National Archives of Korea 30 Daewangpangyo-ro 851beon-gil, Sujeong-gu, (13449)2 Seongnam-si, Korea

## Abstract

When a fire strikes libraries or archives, physical deterioration of the paper is so severe that its chemical properties are often regarded as less important. However, knowledge of the chemical changes of the papers upon nearly burning is necessary to design a subsequent conservation treatment. In the present study, we have examined a rag paper object, which partially survived a fire, and analysed its chemical properties by various methods. The polymeric state of cellulose, as well as its low-molar mass degradation products, were assessed. Comparison to an identical, unharmed duplicate provided a more profound understanding of the changes caused by the fire. Light scattering analysis revealed conformational changes of the cellulose molecule after high-temperature impact, and a chemical cross-linking was observed. In our study, we found the integrity of cellulose to depend on the temperature profile induced by the fire. The low thermal conductivity of cellulose protects the material even in close proximity to the burned edges.

## Introduction

In September 2004, a devastating blaze wreaked havoc on the Duchess Anna Amalia Library in the Thuringian city of Weimar, Germany. A large set of cultural treasures of prime importance in Germany was destroyed. The fire was caused by the defective electrical insulation, resulting in a complete loss of 50,000 volumes and damage to more than 118,000 volumes^1^. The need to minimise physical damage and to hold complete and partial losses of books and manuscripts appeared so pressing that conservation treatments had been applied before some basic understanding was gained of what happened to the paper during the fire. Even if some books and manuscripts managed to escape severe damage, they were affected by chemicals from fire extinguishers, mainly water in this case. The remedial conservation treatments for the objects surviving the fire included simple washing with water and/or reinforcing with supporting substrates, such as Japanese paper^1,2^.

What fire does to historic book paper under the actual conditions of a real fire event has not been studied in detail, let alone well understood. The same applies to what extent the conservation treatments saved or at least improved the fire-damaged paper. Looking at the photos of the damaged books and also the fire-damaged sample material (see Methods and materials section), it was evident that the outside of a book block was charred or completely lost, suggesting the area was directly burned by the fire. On the other hand, the more central areas of the book, approx. 5 cm away from the blackened edge, survived relatively well without distinctive visual change, thanks to the low thermal conductivity of paper^3^ (κ ~ 0.105 W m^−1^K^−1^). The char layer, which is able to protect interior parts of burning timber to some extent from heat^4^ has a thermal conductivity similar to that of paper^5^ and may play a similar role if books are densely packed in shelfs All books that were eventually rescued had been exposed before to the conditions of the room on fire, which involved extremely high temperatures, aggressive gases and mechanical stress due to collapsing supports. In general, fire in closed systems passes through several stages: ignition, growth, flashover, fully developed and decay^6^. When a paper fire reaches flashover, which may take no more than a few minutes after ignition according to fire time-temperature curves, gas temperatures can reach up to 500–600 °C^7,8^. A simulation of a fire on an indoor bookshelf containing various archival and library materials showed the fire to reach 750 °C and higher at the top of the shelving unit after only 15 min^9^. High temperature-induced degradation of paper is a physiochemical process. Suzuki *et al*.^10^ examined physical changes of cellulose filter paper during flame spread under the microscope showing the formation of a colour gradient, black char-yellow-white as well as shrinkage in a heterogeneous way, which was exactly what we observed from the burned papers. Lignocellulosic materials, such as wood or paper, undergo various chemical reactions during pyrolysis depending on the conditions: dehydration, depolymerisation, fragmentation, cross-linking, aromatisation, and so on^11,12^. Oxidation evidently plays an important role in the ignition process of wood^13^. Apparently, numerous products and intermediates from cellulose, such as oligomers and monosaccharides, an indicative pyrolytic product, levoglucosan and aromatic compounds from cyclisations, etc., are involved in the complex chemical reactions during pyrolysis of biomass^14,15^.

The average room temperature during the blast at Anna Amalia Library was well over 200 °C, which is similar to the ignition temperature and starting temperature of thermal decomposition for cellulosic materials^11,16^. Several questions arose concerning the book paper that survived the fire; for instance, how much the cellulose integrity suffered from the high-temperature impact, whether thermal stress was accompanied by oxidative stress, and how these factors affected the long-term stability of the material. As residual degradation products remained in the paper, their influence on paper stability was also unclear.

To answer the questions, we obtained historic books made of rag paper, which in part survived the fire of the Anna Amalia Library. The chemical changes brought about by the fire, and the subsequent extinguishing was comprehensively analysed by a combination of techniques. To better compare the information to the naturally aged state of the paper before the catastrophic event, we correlated the data to the ones from an identical copy of the same book unaffected by fire.

Special emphasis was placed on the presence of low-molar mass degradation products. To address this issue, we applied both established and newly developed chemo-analytical techniques. Extracts obtained with various solvents were analysed by gas chromatography-mass spectrometry (GC-MS)^17^, and by a recently developed analytical tool, namely desorption electrospray ionisation-mass spectrometry (DESI-MS)^18,19^, which was employed to map polyaromatic hydrocarbons (PAHs) on the surface of the burned area. We have also investigated the effect of different washing treatments (water, ethanol) on cellulose stability after applying accelerated ageing. Based on the experimental results, paper stability and degree of damage were assessed, and recommendations for conservation measures were issued.

## Results and Discussion

### Cellulose integrity

**Figure 1:**
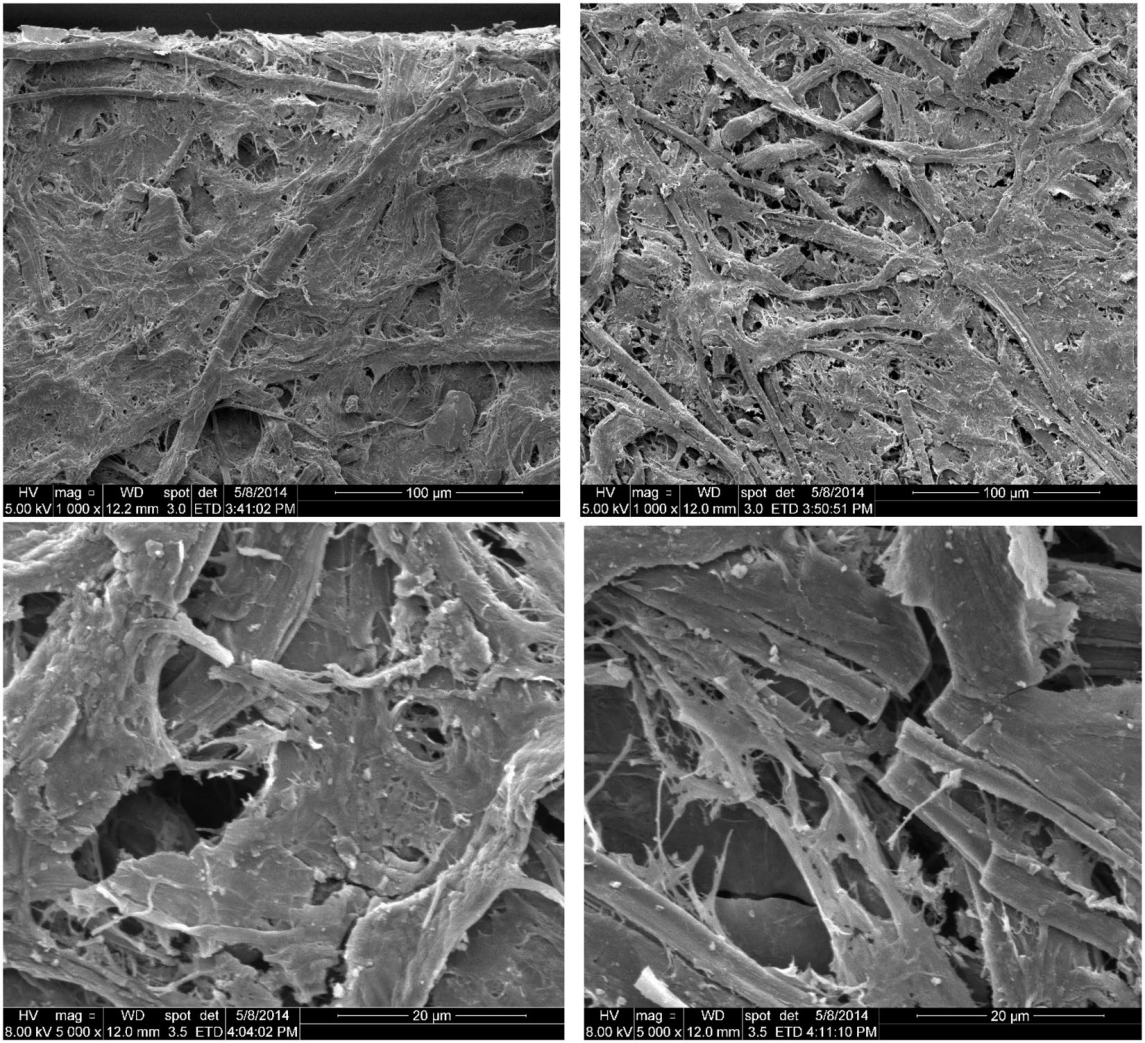
SEM images of the reference paper (not-burned) and the burned paper. Top left: overview of the reference paper. Top right: overview of the burned paper. Bottom left and right: details of the burned paper sample.

The surface of the black broken sample of the burned paper being examined by scanning electron microscopy (SEM) under a low magnification showed a well-retained fibre network although the surface looked somewhat brittle and disturbed by small broken fragments compared to that of the reference paper (Fig. 1 top). The fibre networks on the surface of the burned paper, however, looked carbonised when a higher magnification was applied. Additionally, the broken fibres exhibited sharp edges indicating limited paper strength (Fig. 1 bottom).

**Figure 2:**
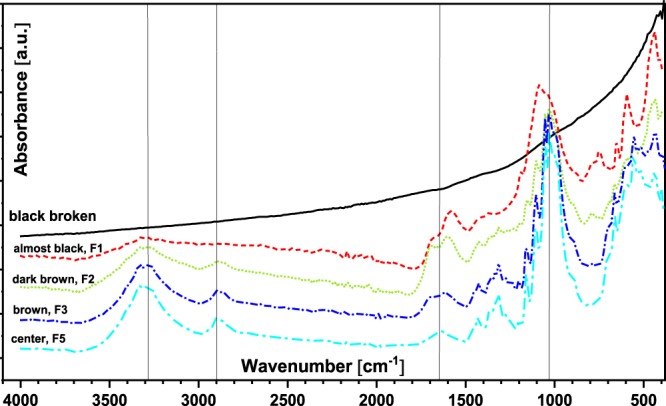
FT-IR spectra of the different areas of the burned paper depending on the extent of damage.

Infrared (FT-IR) analysis of various spots of the burned paper provides a good overview of the structural changes of cellulose in the paper, depending on the extent of damage by the fire. The center of the paper (F5) exhibits typical absorption bands of cellulosic substrates (Fig. 2): broad medium-strong OH vibration at 3250–3400 cm^−1^, CH stretching at 2850–2950 cm^−1^, OH bending 1630–1640 cm^−1^ (possibly overlapped by amide I stretching due to the presence of gelatin as a typical sizing agent of rag paper), CH_2_ at 1430 cm^−1^, CH, OH, CH_2_, CH-bending, OH-bending at 1370–1200 cm^−1^, C-O-C anti-symmetric stretching at 1160 cm^−1^, anti-symmetric in-phase ring stretching at 1105 cm^−1^, C-O-C and C-O at 1060-1030 cm^−1 ^^12^.

Preservation of the IR features of cellulose in the central area of the book paper does not necessarily imply that no degradation took place at all. Oxidation of cellulose is often hard to identify from FT-IR as carbonyls in cellulose easily form hydrates and hemiacetals/hemiketals and hence do not show the distinct bands for sp^2^-keto and sp^2^-aldehydes as expected^20^. The OH band at 1640 cm^−1^ is usually a water adduct present in neat, non-oxidized cellulose as well. Presumably, the extent of oxidation was not significant enough to be represented as a clear separate band. The IR spectrum of the brown area (F3) shows more indication of structural changes. A stronger absorption band at 1700 cm^-1^ is clearly visible, which can be attributed to increased carbonyl or carboxyl motifs. A slight shift of the 1640 cm^−1^ band towards lower wavenumbers was also recognisable. The absorption at 1625 cm^−1^ indicates the formation of carboxyl groups in cellulose, in addition to the conjugated C=C stretching band, occurring due to dehydration at 200–240 °C during cellulose pyrolysis^21^. Other carbohydrate features in the IR spectrum still seem to be not significantly influenced by the fire. When the dark brown area (F2) is analysed, the absorption bands at around 1700 cm^−1^ and 1600 cm^−1^ become more pronounced, and all characteristic bands for cellulose become very broad. The absorption band at 1700 cm^−1^ is weaker in the almost black area (F1) while the band at 1600 cm^−1^ is still relatively intensive and shifted to a slightly lower wavenumber. Comparison of these data to that of Tang and Bacon^199^ indicated that the dark brown area (F2) of the burned paper could have well-experienced temperatures above 280 °C but below around 400 °C because the conjugated C=O stretching at 1700 cm^−1^ is still intense since thermal decarbonylation had not yet occurred. The almost black area (F1), on the other hand, shows dominant conjugated C=C bonds indicating the temperature of the fire was close to 400 °C. The black broken part offered no specific absorption bands, demonstrating that all structural FT-IR features of cellulosic paper were lost although the fibrous macroscopic structure still looked intact (Fig. 1).

Biomass pyrolysis undergoes four stages in general: moisture evolution, hemicellulose decomposition, cellulose decomposition, and eventually lignin decomposition^22^. To further assess the temperature ranges the burned paper could have been exposed to, we determined the carbohydrate compositions by GC-MS after acid methanolysis, which is a sensitive way to address the more vulnerable hemicellulose fraction^23^. Thermal decomposition of lignocellulosic natural fibres starts with the decomposition of hemicellulose at around 250 °C followed by cellulose at around 340 °C^24^. Hence, xylose can serve as a good indicator monosaccharide to roughly determine the extent of thermal decomposition^25^.

**Figure 3:**
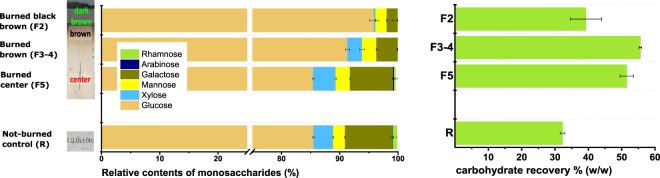
The carbohydrate compositions of the different spots of the burned paper and the not-burned reference paper determined by acidic methanolysis (left) and by total hydrolysis (right).

Taking advantage of the different thermal decomposition characteristics of cellulose and hemicellulose, which are the major composition elements of the original paper, we estimated the temperature ranges the burned papers were exposed to. The central area of the burned paper (F5) exhibited a monosaccharide composition similar to the not-burned reference paper, sharing the fingerprint region of the hemicellulose fractions (Fig. 3, left). Hence, the temperature of the central area did not reach more than 250 °C as the content of hemicellulose stayed unchanged.

By contrast, the brown area of the burned paper (F3-F4) showed significantly lower contents of hemicelluloses, and the relative decrease of xylose is especially distinctive. Therefore, the brown area of the burned paper must have been exposed to temperatures higher than 250 °C, which corresponds to the result from FT-IR analysis. The temperature would not have exceeded around 340 °C extensively since cellulose was not significantly decomposed (Fig. 3 right). The dark brown area of the burned paper (F2) showed decreases in both hemicellulose content and the glucose yield, and the temperature of this area reached around 340 °C and above.

**Figure 4:**
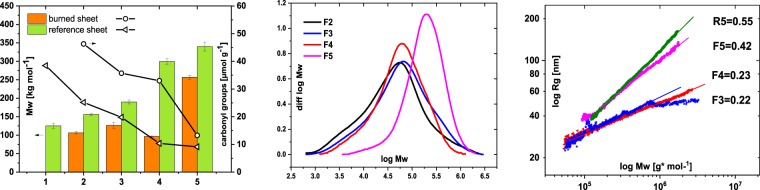
Mw and MMDs of the different areas of the burned paper, depending on the extent of damage by fire. Left: Molar mass and the total contents of carbonyl groups. Middle: Molar mass distributions. Right: Conformation plots showing the fire-induced chemical cross-linking. Numbers correspond to the respective slopes.

Although the temperature ranges of various spots of the burned paper could be roughly estimated, the molar mass profiles of the cellulose chain in different areas are of interest since they are one of the key factors to be considered when a conservation strategy is established. We analysed the molecular mass distribution (MMD) of cellulose and the total carbonyl group contents of the burned paper by gel permeation chromatography coupled to multi angle light scattering (GPC-MALLS)-fluorescence labeling^26^. The central area of the burned paper (F5) showed an weight average molar mass (Mw) about 15% lower and a carbonyl group content about 30% higher than the reference paper that had 304 kg·mol^−1^ (DP 1870) and 9.1 µmol·g^−1^ carbonyl groups (Fig. 4 left). While that central area showed no significant differences from the reference paper according to visual appearance, IR spectrum or carbohydrate compositions, it evidently still suffered a slight but detectable oxidation and depolymerisation of cellulose. This trend enhanced towards the edges of the paper with increasing brown discolouration (F4). Hydrolytic degradation and oxidation increased drastically, and cellulose in this area showed about 2.6 times lower Mw and 2.5 times higher carbonyl group contents than in the center (F5). Looking at the MMD of F4 compared to that of F5 (Fig. 4 right), the entire MWD of the cellulose in the F4 area shifted towards the lower MMD values and became more dispersed, which is a typical pattern of cellulose degradation by hydrolysis. This trend stopped in the dark brown area (F3). F3 gave rise to an increase in Mw compared to F4, indicating a degradation mechanism other than chain scission taking place: cross-linking of the cellulose occurred since the MMD of F3 was shifted to higher molecular weights than that of F4.

The conformation plot, which is a double logarithmic plot of the radius (here r_g_ - radius of gyration from light scattering) and the molar mass of dissolved polymer molecules, strongly suggested a cross-linking of cellulose (Fig. 4 right). The typical rag paper yields a slope of 0.55 whereas, in the heat-exposed paper, a slope of 0.42 down to 0.22 was observed. Hence, the conformation plot showed that heat-exposed cellulose in all areas contained more compact macromolecules depending on temperature with higher heat impact causing more highly cross-linked molecules. Towards the edges of the sheet, the cellulose molecules, when dissolved, were getting increasingly compact: a smaller slope in the conformation plot means similar molar mass at a smaller radius.

This effect seemed to be independent of the depolymerisation degree; hence, cross-linking and depolymerisation occurred simultaneously. While the oxidation significantly increased in samples with higher exposure temperatures (see Fig. 4), the degree of cross-linking and the degree of polymerisation stay largely constant. According to Chaiwat *et al*.^27^, cross-linking of cellulose is more pronounced during slow pyrolysis than during fast pyrolysis. Therefore, the border between F2 and F3 seems to indicate whether the fire flame directly damaged the paper or not. In other words, the dark brown area in F2 was affected by the fire directly causing a process similar to fast pyrolysis with a rapid temperature increase, whereas F3, right next to F2, was degraded rather by heat transfer from F2 resulting in a lower heating rate and subsequently slightly higher cross-linking. Significant depolymerisation of cellulose with little cross-linking in F4 was due to an even lower temperature than in F3.

### Low-molar mass compounds

**Figure 5:**
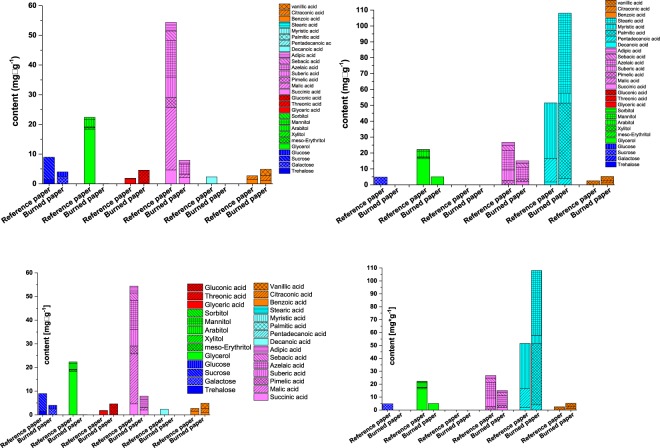
Compounds identified by GC/MS after extraction of the burned paper. Left: Soxhlet water extraction, Right: Soxhlet ethanol extraction.

Low-molar mass compounds play a special role in paper ageing as they accumulate in the paper matrix. These small molecules comprise several acids, aldehydes and ketones, mainly originating from carbohydrate degradation. After exposure to extreme heat, the formation of very non-polar PAHs is expected as well. The entrapment of the degradation product cocktail within the paper accelerates paper ageing and hence cellulose degradation. The question in the present case was whether the fire had an influence on the generation and the typical pattern of low-molar mass degradation products and whether those fragments needed to be removed by, e.g., a washing step to stabilise the paper matrix. To analyse the polar degradation products in paper, we performed a Soxhlet extraction with different solvents followed by GC-MS analysis of the extracts according to a recently developed derivatisation method based on ethoximation and silylation. This method allows for quantification of a large number of carbohydrate-born acids, aldehydes and degradation products. Figure 5, left, shows the results from water extraction. The compounds are grouped into different classes of compounds for clarity. Figure 5, right, gives the compounds in the ethanolic extracts. Table 1 summarises the masses extracted with the different solvents as determined by GC-MS. Overall, the burned paper contained only 23–25% of the extractables of the non-burned reference after water and chloroform extraction. In the first case, the decrease is mainly attributed to losses in monomeric sugar alcohols and sugars. A reduction in long-chain fatty acid accounts for the loss of extractives with chloroform as the solvent. For the burned paper, only the ethanol extract shows 20% more extracted material.

**Table 1:** Extracted low-molar mass compounds by different solvents (N = 2).

	Water		Ethanol		Chloroform	
Analyte	Reference paper	Burned paper	Reference paper	Burned paper	Reference paper	Burned paper
total (mg/g)	92.6	21.4	107.8	133.4	123.2	31.5

Generally, we assume, that the 40% loss in small fragments is simply due to heat degradation, i.e., burning.

**Figure 6:**
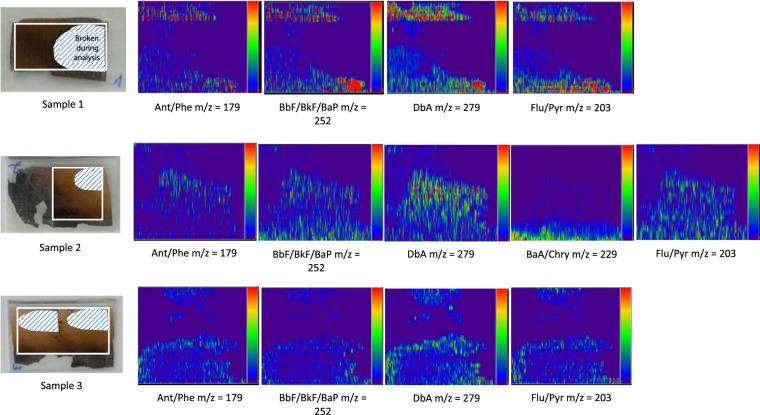
Mapping of PAHs on the surface of the burned paper by DESI-MS: Ant – Anthracene, Phe – Phenanthrene, BaA - Benzo[*a*]anthracene, BbF-Benzo[*b*]fluoranthene, BkF - Benzo[*k*]fluoranthene, Flu - Fluoranthene, Pyr - Pyrene, DbA - Dibenzo[*a,h*]anthracene, Chry- Chrysene, BaP - Benzo[*a*]pyrene.

Desorption electrospray ionisation mass spectrometry (DESI-MS) provides a spatial resolution (mapping) of the contents of low molecular weight compounds across paper surfaces. In the present study, we selected PAHs to be analysed on the sample surface. For comparison, we chose the 16 PAH standards applied to Whatman No. 1. The data acquisition was carried out in scan mode to obtain better resolution while generating images. PAH mapping was successful, with the minor limitation that certain PAHs (Fig. 6) could not be distinguished since they essentially show the same monoisotopic mass (Table S1).

After the successful method setup with PAH standards, we analyzed differently burned samples (F1-F5). Here the DESI method showed its benefits because even samples being sensitive to physical manipulation can easily be analysed under ambient ionisation conditions. However, even with this mildest method, some parts of the critically degraded sample material broke during analysis. PAHs were detected in all samples, which is consistent with the literature^28^. The distribution of the PAHs increased towards the more burned edges, i.e., with progressing thermal degradation (pyrolysis) as expected (Fig. 6).

In summary, DESI-MS appears to be a suitable method for detecting PAHs on thermally treated or fire-damaged cellulosic sample material as shown in Fig. 6.

### Fire damage - long-term effect on cellulose integrity

After analysis of the burned papers, the question that still needed to be answered was which conservation strategy has to be established for the burned paper. Would a mechanical stabilisation of the partially fragile sheets be sufficient, or would it further trap degradation products? Could a washing step be beneficial to increase the paper stability for future storage? It was clear that the dark brown paper area, like in F2, required mechanical support since it was too brittle to be handled and most likely would not have survived additional treatment steps.

**Figure 7:**
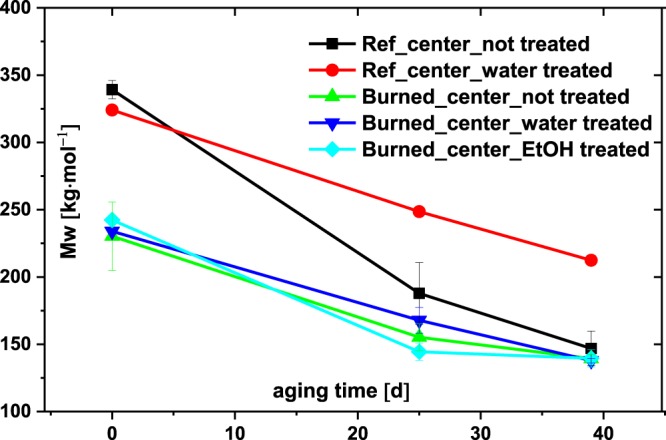
Mw of the samples (N = 2) with and without washing treatment prior to accelerated aging at 80 °C and 65% RH.

Therefore, we focussed for this part of the study on the relatively intact areas (F3-F5) and investigated their long-term stability after accelerated ageing at 80 °C and 65% RH as well as in comparison to the reference paper. A simple washing step with water or ethanol was tested to see whether the long-term stability was improved by removing degradation products induced by the fire. As seen in Fig. 7, the central area of the burned paper (F5) decreased its Mw regardless of any prior washing step whereas the reference paper strongly benefited from the washing as it slowed down the degradation process. This effect is known to conservators; the washing-out of degradation products, mainly acids, can have a positive effect on long-term paper stability.

The burned paper, on the other hand – even the central area, which was less affected by the fire – did not benefit at all from the washing treatment, no matter whether water or ethanol was used. Further degradation proceeded to about the same final degree of polymerization (DP). Interestingly, the final molar mass of the cellulose after 40 days of ageing was similar for the non-washed reference paper and the differently treated burned papers.

The substances in the reference paper that were washed out and thus could not further trigger degradation upon accelerated ageing were not anymore contained in the burned papers. Eventually, the burned paper degraded with a similar speed compared to the unburned reference, just starting from an initially lower DP (Fig. 7). In addition, the number of substances extractable by water from the burned paper was much less (1/4) compared to the reference paper (*see 3.2*). Thus, the effect of water-washing for the burned species is thus very limited, and a corresponding conservation treatment cannot be justified.

## Conclusions

In the present study, a detailed investigation of historic book paper exposed to a fire and the subsequent extinguishing processes has been carried out in comparison to an unharmed duplicate not exposed to the fire blast. The heat gradients in the book from the central parts, which seemed still largely intact towards the completely damaged and burned edges, were estimated by characterizing the molecular state of the cellulose using multi-detector GPC. For the first time, a heat-induced cross-linking was demonstrated as a consequence of heat exposure in addition to the expected thermal chain degradation. The cross-linking increases with increasing temperature towards the edges and eventually renders burned papers more brittle. A slight cross-linking as demonstrated for the middle of the book pages may even have positive effects on stability.

As well, degradation products, sugar composition and formation of PAHs were compared. The low heat transfer due to the low thermal conductivity of paper^3,29^ with ~0.05–0.1 W/m K preserves areas, which are already in very close proximity of 2–3 cm to extreme heat conditions. Typical low-molar-mass degradation products are diminished by the heat so that a washing treatment is neither detrimental nor beneficial. The removal of polar degradation products in the identical reference book had a significant beneficial effect on the ageing behaviour. As expected, PAHs formed in the black areas towards the completely burned outside parts of the sheet, which eventually only contained carbon, but interestingly, with well-preserved cellulose fibre morphology.

## Methods and Materials

### Chemicals

*O*-Ethylhydroxylamine hydrochloride, *N,O*-bis(trimethylsilyl)trifluoroacetamide (BSTFA), trimethyl-chlorosilane (TMCS), 4-dimethylaminopyridine (DMAP), acetic acid (98+%) and EPA 610 Polynuclear Aromatic Hydrocarbons Mixture (certified reference material) was purchased from Sigma-Aldrich/Fluka (Schnelldorf, Germany). Ethyl acetate (99.8%) was obtained from Merck (Darmstadt, Germany). Pyridine (99.5%, extra dry) was from Acros Organics (Geel, Belgium), methanol (99.99%) from Fischer Scientific (Loughborough, UK).

### Sample materials

**Figure 8:**
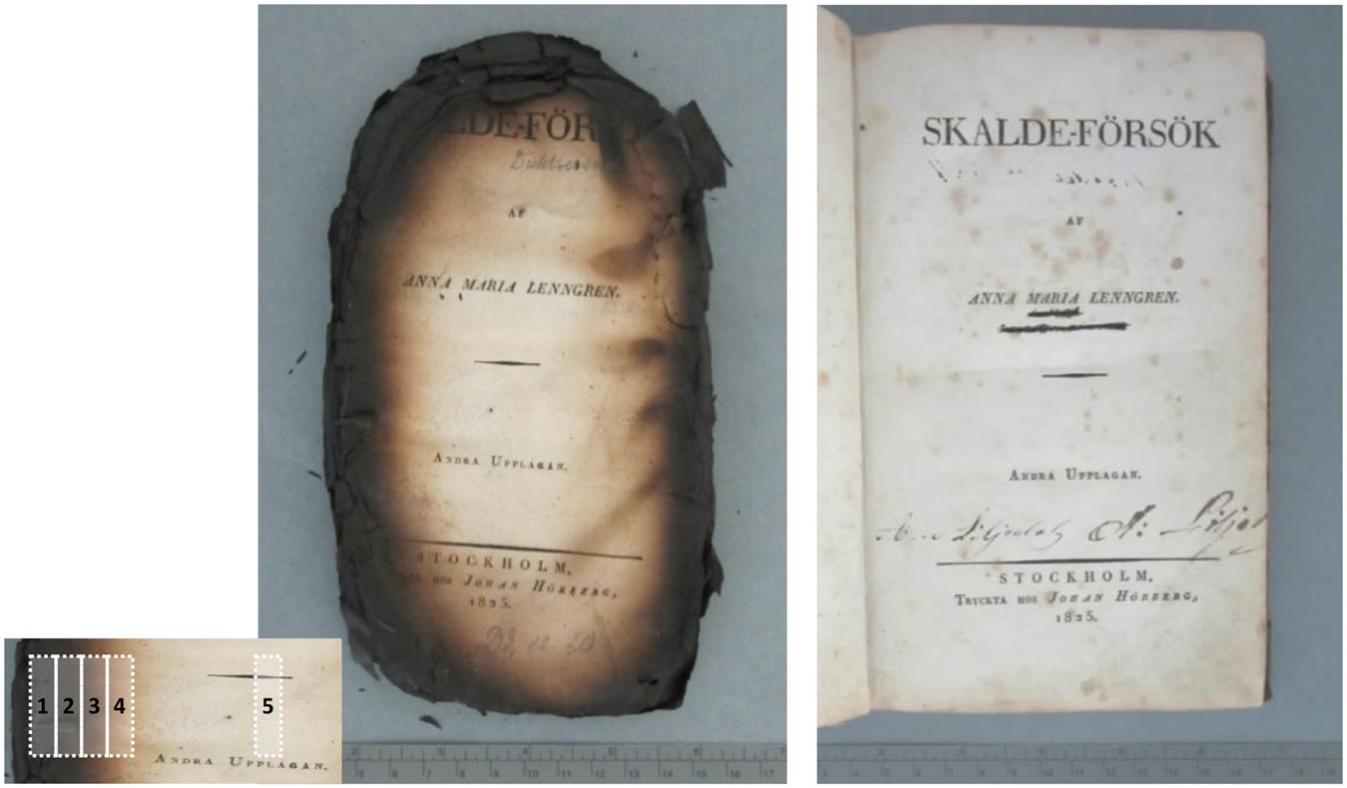
Book papers analyzed in the study. Left - burned book by the fire at the Duchess Anna Amalia Library in 2004. The lower left image indicates where the samples were taken (5-center; 4- light brown; 3-brown; 2-dark brown; 1-black brown). Right – not-burned identical book.

A book damaged by the fire at the Duchess Anna Amalia Library in 2004, and a not-burned identical book were contributed by the Anna Amalia Library. The book (‘Skalde-Försök’ by Anna Maria Lenngren, 2^nd^ edition, Johan Hörberg: Stockholm), made of rag paper, was published in 1825 (Fig. 8).

Various instrumental analyses of the burned paper and the not-burned reference paper were performed. In case of the burned paper, the areas analysed varied depending on the extent of damage are indicated in Fig. 8, left. When specific areas were analysed, their data were presented with the letter “F” (Fire) and “R” (Reference) for the burned paper and the not-burned reference paper, respectively.

### Sample preparation for GPC and GPC conditions

Carbazole-9-carbonyl-oxy-amine (CCOA) labeling of carbonyl groups was performed as described earlier^26^. After labeling the samples, dissolution in *N,N*-dimethylacetamide/lithium chloride 9% (w/v) (DMAc/LiCl) was achieved after a solvent exchange at room temperature. Cellulose analysis was performed with a GPC (gel permeation chromatography) MALLS (multi-angle laser light scattering) fluorescence detection system, which yields the molecular weight distribution (MWD) in addition to the profiles of oxidised groups relative to the MWD.

The GPC system consisted of a TSP FL2000 fluorescence detector for monitoring the CCOA label, a MALLS detector, (Wyatt Dawn DSP, Wyatt Inc. Santa Barbara, USA) with an argon ion laser (λ_0_ = 488 nm) and a refractive index detector (Shodex RI-71). Four serial GPC columns, PLgel-mixed ALS, 20 µm and 7.5 × 300 mm (Agilent, Waldbronn, Germany) were used as the stationary phase. A degasser (Dionex DG-2410), autosampler (HP 1100), pulse damper pump (Kontron pump 420), and column oven (Gynkotek STH 585) were also part of the system.

The operating conditions of the GPC were as follows: 1.00 mL/min flow rate, 100 µL injection volume, 45 min run time and λ_ex_ = 290 nm and λ_em_ = 340 nm for fluorescence detection of the CCOA label. DMAc/LiCl (0.9%, w/v) was used as an eluent after filtering through a 0.02 µm filter.

The data were evaluated with standard Chromeleon, Astra, and GRAMS/32 software.

### GC-MS

Withdrawal of sample material was performed by Soxhlet extraction with water, ethanol or chloroform.

An aliquot of the sample material was lyophilised in a 1.5 mL GC-vial. Subsequently, 100 µL of β-methyl galactopyranoside acting as an internal standard, with 2 g/L dissolved in water, was added followed by lyophilisation. 200 µL pyridine was added to the dry sample. 200 µL of a solution containing 40 mg/mL *O*-Ethylhydroxylamine hydrochloride in pyridine was added followed by heating at 70 °C for one hour. Finally, the sample was allowed to cool to room temperature. Once cooled, 200 µL of pyridine containing 1.5 mg/mL DMAP and 200 µL of BSTFA containing 10% (v/v) TMCS were added followed by heating at 70 °C for two hours. After cooling down to room temperature, 600 µL ethyl acetate were added prior to analysis.

GC-MS analysis was carried out on an Agilent 7890 A gas chromatograph coupled with an Agilent 5975C-triple axis mass selective detector (MSD; Agilent Technologies, Waldbronn, Germany). The GC was equipped with an Agilent split/splitless inlet which was connected to a DB5-MS (5% phenyl- 95% dimethyl-polysiloxane phase) column (30 m × 0.25 mm i.d. × 0.25 µm film thickness; J&W Scientific, Folsom, CA, USA). The split/splitless inlet was operated under the following conditions: constant column flow; 0.9 mL/min using helium carrier gas, injector; splitless mode at 260 °C. Oven temperature gradient profile: 50 °C (2 min), 5°C/min to 280 °C (20 min) and back to initial. The MSD was operated in EI-mode at 70 eV ionisation energy and 1.13 × 10^−7^ Pa. Ion source temperature: 230 °C, quadrupole; 150 °C, transfer line; 280 °C. Aliquots of 0.2 µL were injected by a RTC-PAL autosampler, which was controlled by Chronos software v.4.4.2.0 (Axel Semrau, Spockhövel, Germany). Data analysis was carried out by Agilent MSD ChemStation (v. G1701EA E.02.02.1431).

### DESI-MS

The sample measurement was carried out on a Thermo LTQ (LTQ XL™ Linear Ion Trap Mass Spectrometer, Thermo Fisher Scientific, Waltham, Massachusetts, USA). The DESI ion source was built in-house based on a manual Omnispray ion source (Prosolia Inc., Indianapolis, Indiana, USA) equipped with a sustom-made X/Y-axis moving stage (MTSys Inc., Linz, Austria). The following instrumental parameters were used throughout the experiments: Spray solvent flow rate 5° µL*min^−1^ (methanol with 1% acetic acid); N_2_-nebuliser gas flow rate 1 L*min^−1^. Spray angle, tip to surface distance and sample to transfer capillary distance were set to 60°, 3 mm and 0.5 mm, respectively.

A spray-head consisting of fused silica capillaries and a Swagelock-T-piece (Swagelock Inc., Solon, Ohio, USA) as described in Ellis *et al*.^30^ was used with inner capillary: 50 µm I.D., 150 µm O.D.; outer capillary: 250 µm I.D., 320 µm O.D.; scan velocity: 200 µm*s^−1^. Mass spectra were acquired in positive ion modes from 110 to 350 m/z with an extraction width of ± 0.5 m/z. The capillary temperature was set at 275 °C and 4 kV spray voltage was used throughout the experiments.

### Preparation of standards

EPA 610 PAH mix standard containing the compounds shown in Table 1 was used for verification of the target substances. After 1:10 (v/v) dilution of the standard mix in methanol, an aliquot was applied to filter paper (Whatman No.1) and air-dried before measurement.

### SEM

A broken black piece of the burned paper and the reference paper were analysed by SEM (FEI Inspect^TM^ S50, Oregon, USA). The measurement conditions are presented on the bottom of each SEM image.

### FT-IR

Infrared spectra of the various areas of the burned paper depending on the extent of the burning were obtained using FT-IR – ATR (Attenuated Total Reflection) (Bruker alpha-P, diamond ATR crystal, Germany). Spectral collection was performed under ambient conditions with the following operating conditions: range 4000-400 cm^−1^; resolution 4 cm^−1^; scan number 128.

### Washing treatment

Approx. 2.5 g of the burned paper (~3 sheets) and 2.7 g of the reference paper (~2 sheets) was washed in a water bath containing 375 mL and 400 mL of extra purified water (0.55 μS·cm^−1^), respectively, for 30 min with gentle shaking. The water-washing step was carried out three times. After removal of excess water between acid-free absorbent blotting papers (KLUG conservation GmbH, Germany), the papers were dried under ambient conditions. Each sample (1.8 g) was also washed once with 150 mL of ethanol (96%, v/v) and dried the same way.

### Accelerated ageing

The accelerated ageing of the samples after the washing steps was carried out at 80 °C and RH of 65% in a climate chamber (Q-LAB Q-Sun Xe-3, USA) based on ISO 5630-3:1996^31^ for 25 days and 39 days.

### Data availability statement

Data are available upon request from the authors.

## Electronic supplementary material

Supplementary Information
