# Supplementary material for: Fire-induced structural changes and long-term stability of burned historical rag papers
Source: Sci Rep. 2018 Aug 13;8:12036. doi: 10.1038/s41598-018-30424-7 (PMC6089898; doi:10.1038/s41598-018-30424-7)
Supplement: Supplementary file 1 — Supplementary Information [file 41598_2018_30424_MOESM1_ESM.docx]

**Supplement - SciRep**

**“Fire-induced structural changes and long-term stability of burned historical rag papers”**

Kyujin Ahn^1,2^, Andreas Schedl^1^, Thomas Zweckmair^1^, Thomas Rosenau^1^, Antje Potthast^1*^

^1^University of Natural Resources and Life Sciences, Vienna, Department of Chemistry, Division of Chemistry of Renewable Resources, Muthgasse 18, 1190 Vienna, and Konrad Lorenz Straße 24, A-3430 Tulln, Austria

^2^Archival Preservation and Restoration Center, National Archives of Korea

30 Daewangpangyo-ro 851beon-gil, Sujeong-gu, Seongnam-si, Korea (13449)2

*corresponding author: Antje Potthast (antje.potthast@boku.ac.at)

**Table S1**: PAH standards detected by DESI-MS.

| PAH | Abbreviation | Formula | Measured ions | Monoisotopic mass |
| --- | --- | --- | --- | --- |
| Anthracene | Ant | C_14_H_10_ | [M+H]^+^ | 178.0783 |
| Benz[a]anthracene | BaA | C_18_H_12_ | [M+H]^+^ | 228.0939 |
| Benzo[b]fluoranthene | BbF | C_20_H_12_ | M^+^ | 252.0939 |
| Benzo[k]fluoranthene | BkF | C_20_H_12_ | M^+^ | 252.0939 |
| Benzo[a]pyrene | BaP | C_20_H_12_ | M^+^ | 252.0939 |
| Chrysene | Chry | C_18_H_12_ | [M+H]^+^ | 228.0939 |
| Dibenz[a,h]anthracene | DbA | C_22_H_14_ | [M+H]^+^ | 278.1096 |
| Fluoranthene | Flu | C_16_H_10_ | [M+H]^+^ | 202.0783 |
| Phenanthrene | Phe | C_14_H_10_ | [M+H]^+^ | 178.0783 |
| Pyrene | Pyr | C_16_H_10_ | [M+H]^+^ | 202.0783 |
